# Factors That Influence a Mother’s Willingness to Preserve Umbilical Cord Blood: A Survey of 5120 Chinese Mothers

**DOI:** 10.1371/journal.pone.0144001

**Published:** 2015-12-09

**Authors:** Haiyan Lu, Yanwen Chen, Qiaofen Lan, Huanjin Liao, Jing Wu, Haiyan Xiao, Carol A. Dickerson, Ping Wu, Qingjun Pan

**Affiliations:** 1 Clinical Research Center & Institute of Nephrology, Affiliated Hospital of Guangdong Medical College, Zhanjiang, Guangdong, China; 2 Department of Anesthesiology & Perioperative Medicine, Georgia Regents University, Augusta, Georgia, United States of America; Indian Institute of Toxicology Research, INDIA

## Abstract

**Background:**

Umbilical Cord blood (UCB), which contains a substantive number of stem cells, could be widely used in transplants to treat a variety of oncologic, genetic, hematologic, and immunodeficiency disorders. However, only a small portion of mothers preserve or donate their UCB in China. The limited availability of UCB has hampered stem cell research and therapy nowadays. To date, no systemic investigations regarding factors that influence a mother’s willingness to preserve UCB have been performed in China. In the current study, we are trying to determine those factors which will provide useful information for national health policy development and will raise awareness of the importance of UCB preservation.

**Methods:**

During 2011 to 2013, 5120 mothers with the average age of 26.1±8.4 years were included in this study. Those mothers participated in a standardized survey. The information gathered consisted of delivery time, occupation, level of education, knowledge of preservation of UCB, willingness to store UCB, and related concerns. The results have been analyzed with SPSS 16.0.

**Results:**

The results showed that first-time mothers showed a greater willingness to preserve their UCB (73.3%) compared to those having their second (48.9%) or third child (40.3%). Mothers who were employed at Government Agencies and Organizations were more willing to preserve their UCB (87.3%) than those employed at factories (62.0%), and those who were unemployed (27.3%). Mothers holding master’s or college degrees were more willing to preserve their UCB (72.5% and 71.1%, respectively) than mothers with high school diplomas (48.7%) or those who only went to preliminary school or middle school (40.7%). The two strongest factors that influenced an unwillingness to preserve UCB were the high cost and concerns regarding the safety of the preservation.

**Conclusions:**

The results showed that mothers with higher education or those having better occupations are more likely to preserve their UCB in China. These mothers have related knowledge and understand the importance of the preservation and they could more readily afford the relatively high cost. The government, clinicians and UCB banks should combine efforts to take measures, such as increasing public knowledge of the importance of UCB preservation and decreasing the high cost for its storage will most likely increase the frequency of UCB preservation which will further benefit stem cell research and therapy.

## Background

Umbilical Cord Blood (UCB) is a small quantity of blood present in the placenta and umbilical cord after childbirth. In the past this powerful resource was considered useless and often discarded. In recent decades, scientists have discovered that UCB contains a high number of hematopoietic stem/progenitor cells [1, 2], which can rebuild the hematopoietic and immune systems. These stem cells have been used to treat various diseases, which has been termed stem cell therapy [3–6]. As a valuable bioresource for stem cell research and therapy, techniques for the collection, storage, and transplantation of UCB are gradually maturing [7–10]. The related policies regarding the use of autologous UCB are also improving [11], which in turn benefits stem cell research and therapy.

The United States has published legislation to promote the preservation and donation of UCB. Obstetricians are required to inform mothers of the value of UCB and of their right to donate or preserve their own UCB for future use. Thus, in the USA, knowledge regarding UCB preservation has improved, and the proportion of mothers that preserve or donate their umbilical UCB has also increased. However, in China, many parents are not provided this knowledge prior to or at delivery. Also, there is typically only one child per family in China, even China has loosen its decades-long one-child population policy since 2013, it is even more critical to preserve UCB from Chinese babies for possible future use. Since many Chinese do not have many siblings, it is not easy for them to find matched bone marrow donors if they require transplantation. The preserved UCB stem cells have many advantages, such as can be immediately available for use to minimize disease progression in the early treatment stages [3–6]. The nature of the autologous stem cells from UCB will also have fewer complications when used in transplants, since the body will identify them as self. Moreover, UCB stem cells are biologically younger and more flexible compared to those from adult bone marrow [1, 2]. Therefore, it is a necessary and worthwhile endeavor to store UCB.

To a certain extent, Umbilical Cord Blood Banking (UCBB) could be referred to as “life banks”. Stored UCB serves as a backup source or insurance for maintaining a child’s health. An investigation was completed in the USA that included more than four hundred twenty-five patients, who were given a questionnaire, and part 1 of the questionnaire contained questions regarding familiarity with the term UCBB. Patients who indicated any awareness of UCBB were included in part 2 of the questionnaire. Here they were requested to answer more detailed questions in order to evaluate their knowledge of UCBB. The results showed that patients, especially ethnic minorities, younger patients, or patients with lower degrees of education, were poorly informed of UCBB. Very few patients received any UCBB education from health care providers, even most patients were expecting to get more information from their obstetrician during their hospitalization. So, the researchers concluded that lack of knowledge and the high expense were the main barriers to UCBB [12].

Currently, no systemic investigations regarding factors that influence a mother’s willingness to preserve UCB have been performed in China, the largest developing country with a population of more than 1,300,000,000 people.

In this study, we investigated the factors that may influence a mothers’ willingness to preserve their UCB by performed oral interviews of 5120 mothers hospitalized at the Maternal and Child Care Service Centre of Xiashan District, Zhanjiang City and Obstetrics and Gynecology of Affiliated Hospital of Guangdong Medical College. This investigation will provide useful information for national health policy development and will raise awareness of the importance of UCB preservation, which may also improve stem cell research and therapy.

## Methods

The inclusion criteria for participation in this study were as follows: 1) From 2011 to 2013, mothers hospitalized at Maternal and Child Care Service Center of Xiashan District, Zhanjiang City and Affiliated Hospital of Guangdong Medical College; 2) Willingness to join our investigation and answer all questions completely; 3) No limits in regards to parity, occupation, or level of education.

All the participants provide their written informed consent to participate in this study, and the ethics committees of the Affiliated Hospital of Guangdong Medical College approve this consent procedure and this study (No. 20100905038). The interview procedure was standardized and carried out in the form of oral questions and answers which can be found in S1 File. The main topics discussed with the interviewee were as follows: 1) Personal information, including delivery time, occupation(s), and level of education; 2) Whether the patient had any knowledge of the preservation of UCB (if not, an elaborate introduction of the principles, meaning and expenses of UCB preservation was provided, especially for use in stem cell therapy); 3) Whether the patient was willing to store her UCB; 4) If the patient wished to save her UCB, record it. If not, what were their greatest concerns: the safety of the preservation? Consider it no use? High cost or other reasons?

## Statistical Analysis

All statistical tests were performed with SPSS 16.0. The quantitative data was expressed as the means ± S.D. Two group comparisons were carried using our independent sample t test. Multiple group comparison was performed using ANOVA. The categorical data was expressed as frequency and percentage, and between groups were compared with chi-square test. P value was considered as statistically significant if it is less than 0.05.

## Results

### Subjects

A total of 5120 mothers with an average age of 26.1±8.4 years were included in this study. Among those mothers, 3232 were pregnant with their first child, 1360 with their second child, and 528 with their third child. The occupations of the mothers included Government Agencies and Organizations (1376), factories (2948), and 796 of the mothers were unemployed. For the levels of education, 752 of the mothers had earned a master’s degree, 2912 of them held college degrees, 512 of them graduated from high school, and 944 of them only went to preliminary school or middle school. All these detailed information can be found in S1 Table.

### Effect of number of children on willingness to preserve UCB

**Fig 1:**
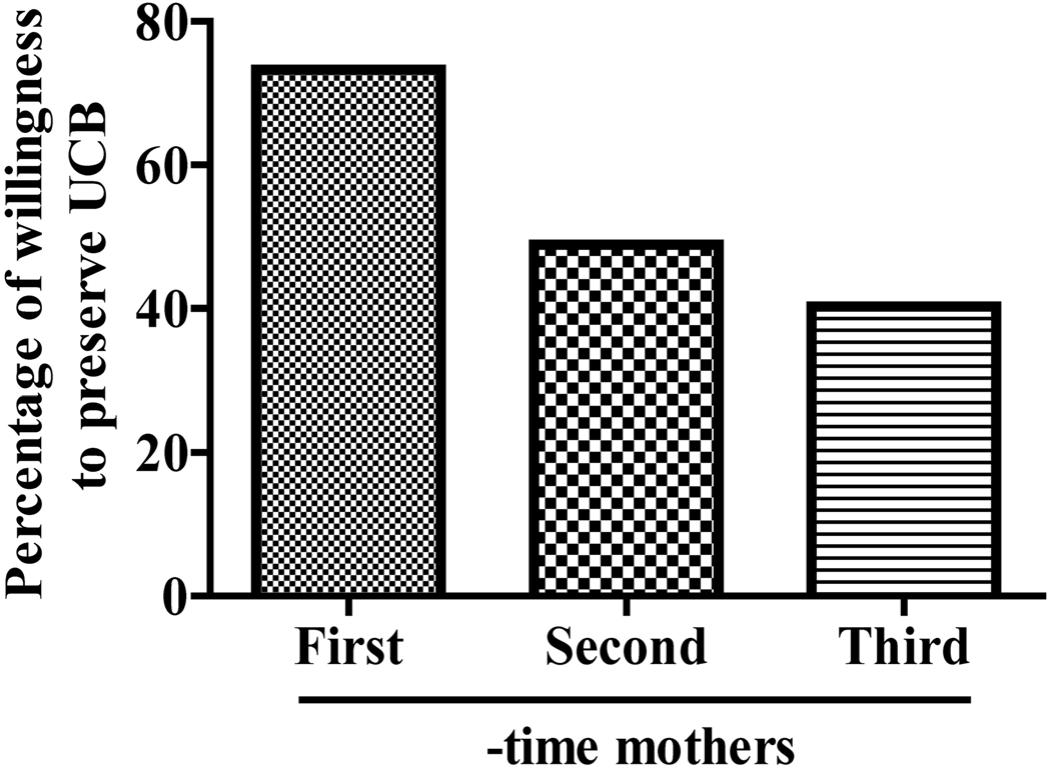
Effect of number of children on willingness to preserve UCB.

According to the survey results, women pregnant with their first child were more willing to preserve their UCB (2369/3232, 73.3%) than those pregnant with their second (665/1360, 48.9%) or third child (213/528, 40.3%) (Fig 1).

### Effect of occupations on willingness to preserve UCB

**Fig 2:**
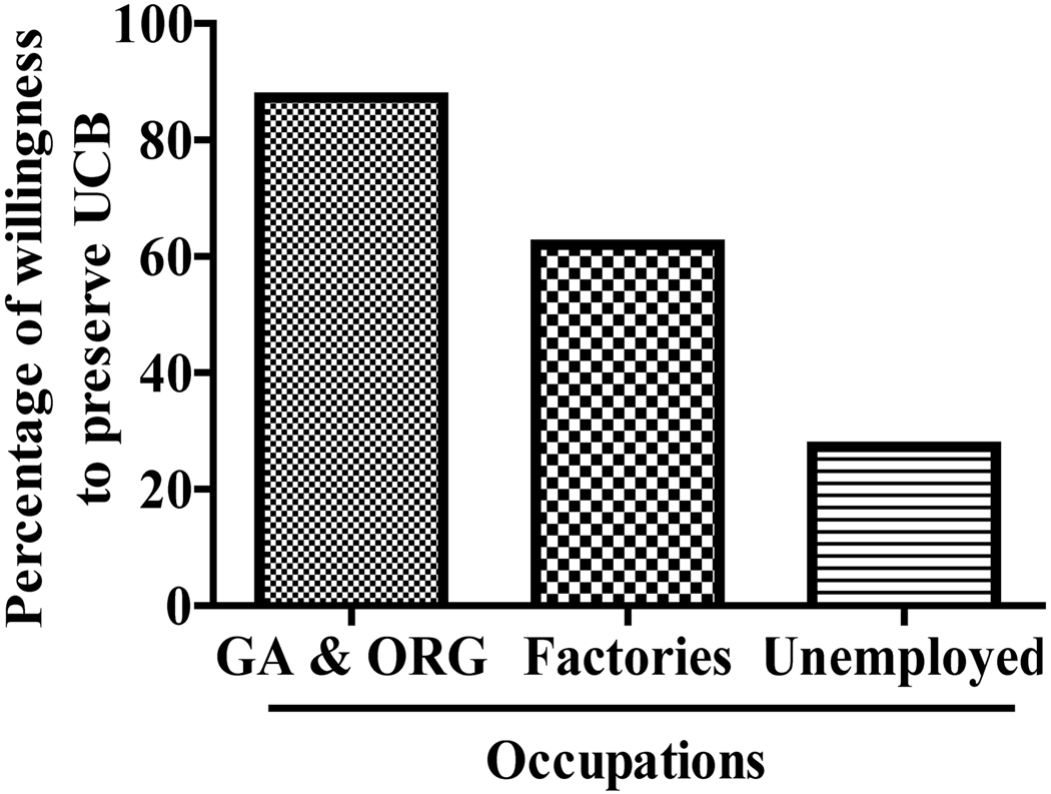
Effect of occupations on willingness to preserve UCB.

The bar graph shows that 87.3% (1201/1376) of the mothers employed at Government Agencies and Organizations agreed to preserve their UCB, while only 62.0% (1829/2948) of the mothers employed at factories wanted to store UCB, and this number reduced to 27.3% (217/796) in those unemployed mothers (Fig 2).

### Effect of education level on willingness to preserve UCB

**Fig 3:**
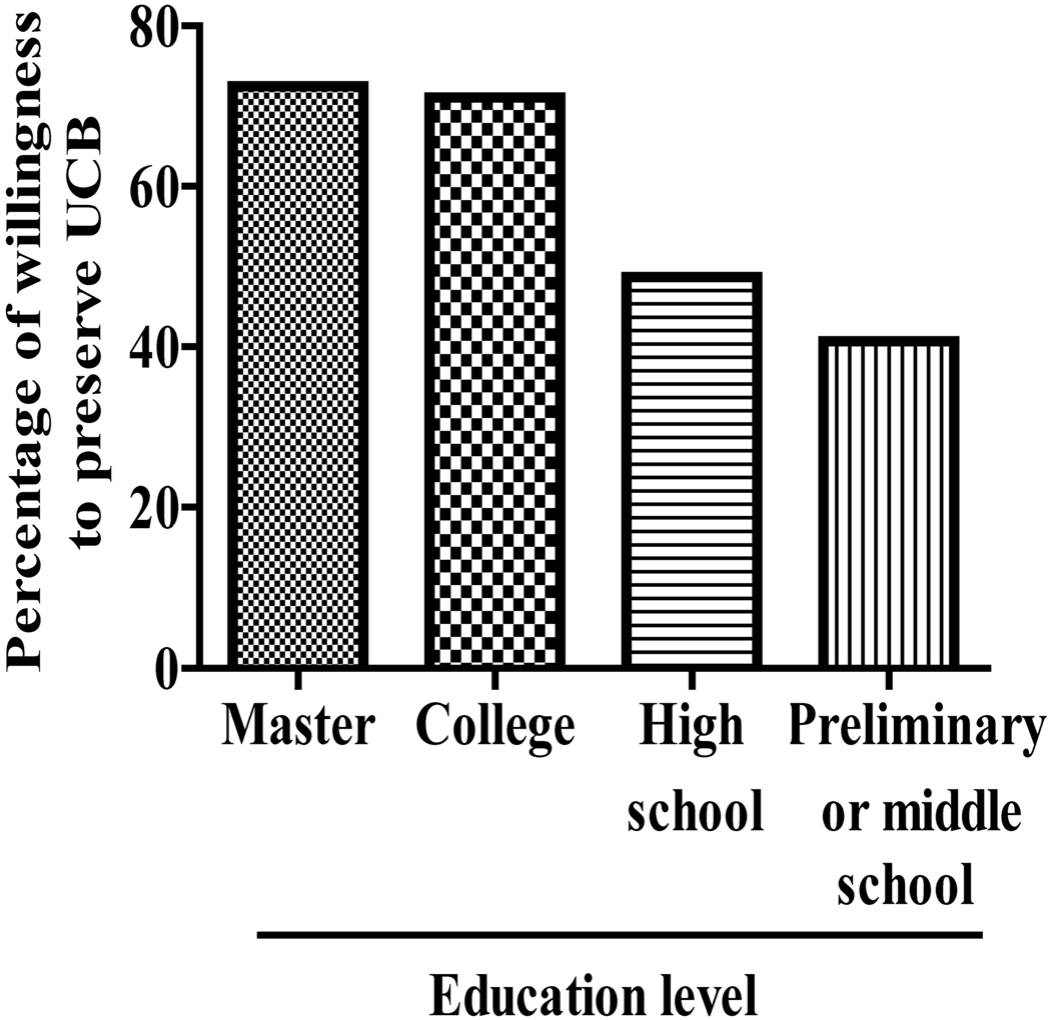
Effect of education level on willingness to preserve UCB.

We determined that mothers with master’s or college degree were more willing to preserve their UCB (545/752, 72.5% and 2069/2912, 71.1%, respectively) than mothers with high school degrees (249/512, 48.7%) and those only went to preliminary school or middle school (384/944, 40.7%) (Fig 3).

### The primary factors influencing willingness to preserve UCB

**Fig 4:**
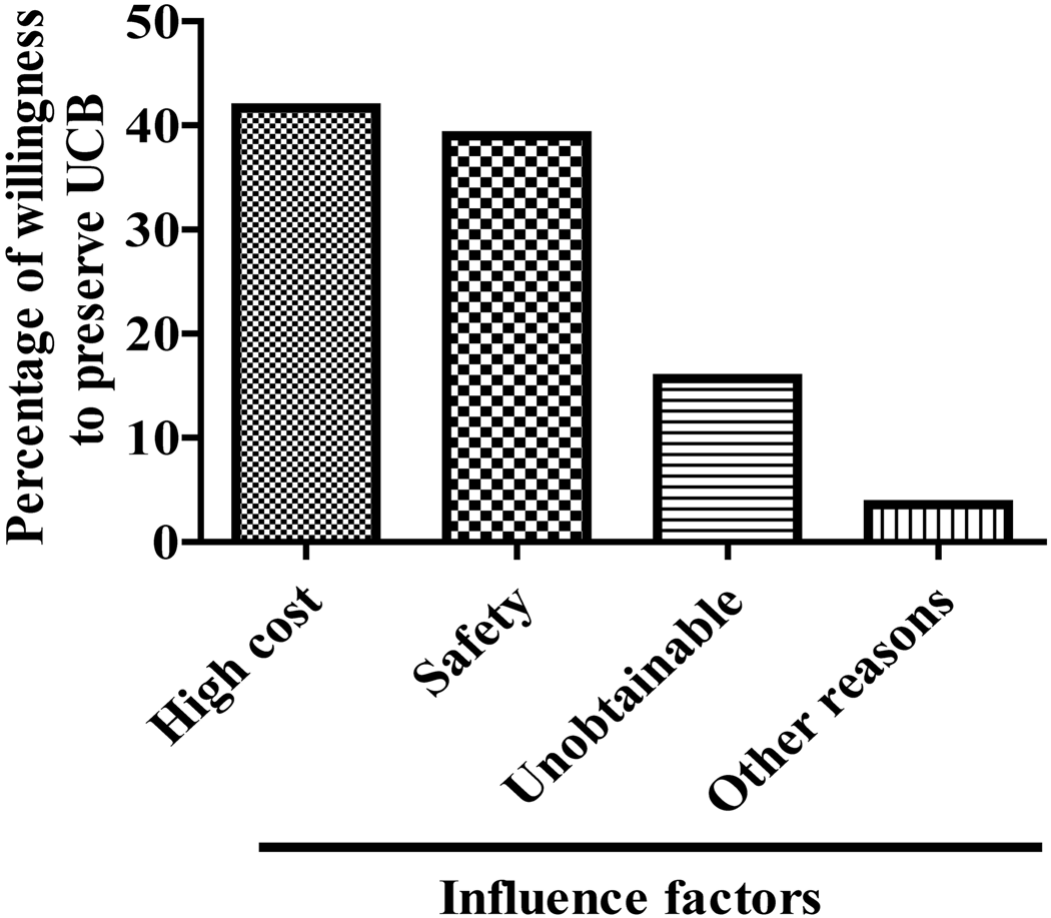
The primary factors influencing willingness to preserve UCB.

As shown, the two strongest factors affecting willingness to preserve UCB were the high cost (41.7%) and concerns regarding the safety of the preservation (39.0%). Some considered UCB preservation unobtainable (15.7%), and only 3.6% expressed other reasons for being unwilling to preserve UCB (Fig 4).

## Discussion

Stem cell research promotes the development of regenerative medicine, which is a medical revolution following drug therapy and surgery [13]. One question asked is: Where can we find a source of “magical” hematopoietic stem cells? For the treatment of childhood leukemia, UCB has become one of the most common sources of hematopoietic stem cells throughout the world, use of these stem cells has become known as stem cell therapy [14]. UCB, which is rich in hematopoietic stem/progenitor cells, is frequently used to as a source of stem cells in order to treat diseases [1, 2]. Stem cells have enormous potential for alleviating the suffering attributed to many diseases which currently have no effective pharmaceutical therapy, such as leukemia, cancer, aplastic anemia and thousands of other refractory and incurable diseases [13]. The use of autologous UCB negates the need to find a bone marrow match and almost eliminates the risk of immune rejection, increases overall survival rate as well [15]. Additionally, UCB can be preserved for a long time. For example, hematopoietic stem cells can be stored for more than 40 years. The technology used for the preservation of UCB has improved dramatically in recent years [16]. Finally, other medical values of UCB may be explored in the future. Also, UCB has the potential for use in organ cloning, repairing senescent tissues and organs, and as a powerful weapon against cancer. Clinical trials using UCB for the treatment of neurological and spinal cord injuries have been started [17, 18].

At present, there are two different kinds of UCBB, public and autologous banks, each of which has advantages and disadvantages [19–21]. Public banks store UCB that is donated by expectant mothers for free and prepares the UCB for queries to find matches for patients in need [20]. Autologous banks store UCB for personal use to ensure that a mother has “life-saving blood” for her child if needed [19].

The results of this study showed that women pregnant with their first child were more likely to be willing to preserve their UCB than those with their second or third child in China. A survey among Greek citizens reported only 6% of the respondents who had children and were in favour of UCB transplantation, had stored or donated UCB [22]. Moreover, mothers employed at Government Agencies and Organizations are more likely to be willing to preserve their UCB than those employed at factories or are unemployed. Additionally, education level plays an important role in the willingness of mothers to preserve their UCB. College-educated mothers are more often willing to store their UCB compared to mothers with a high school education or lower. For Greek citizens with regards to future decisions, 84% of the sample would store or donate UCB [22]. Another study in Istanbul, Turkey also found the majority of pregnant women want to store UCB [23].

In addition, this study found several factors that have the strongest influence on the willingness of mothers to preserve their UCB in China. First, propaganda regarding UCB preservation or uses is inadequate and should be published for public awareness. A survey among pregnant women in Hong Kong found that the majority (78.2% of 1866 women) had no idea that they can use self-stored UCB stem cells. Also, most of the respondents were unclear about which diseases except leukemia are amenable to treatment with UCB stem cells in general. Only 20.3% (of 1866 women) of women knew that stem cells can be get from the Red Cross if their children need hematopoietic cell transplantation [24]. The investigation in Istanbul, Turkey also found that the majority of the participants had a lack of knowledge about stem cells and UCBB [23]. While in Greek citizens, 48% of respondents knew about UCB and had full knowledge about what storage or donation offers [22]. For the source of information or knowledge about stem cells and UCBB, media (newspaper, Internet, television, etc.), and doctors (obstetrician), nurses and midwives were the main source [22–23]. Second, public physicians have limited knowledge regarding the importance of UCB preservation, which can be improved with minimal effort by professional training about the knowledge of stem cells and UCBB. Third, the cost of UCB preservation is high; the state should invest funds in the development of greater public awareness so that UCB will become regarded as a precious resource for public and private health. Private UCBB typically charge around $2,000 for the collection and around $200 a year for storage [19]. Nowadays in China, to store one UCB in Private UCBB require a one-time upfront fee (about 5,000 RMB in China currency, equal to $816.00) and an annual fee (about 500 RMB in China currency, equal to $81.60). Furthermore, the belief that the stored UCB will never be used lead mothers not to store their UCB, to counter this thinking mothers should be informed of the other scientific uses for UCB that are available [25,26]. A study conducted in the USA showed that approximately one in 2,000 children who suffer from malignant diseases can be treated successfully with UCB prior to reaching 20 years of age. Aircraft and fire accidents affect approximately one in a million children. Therefore, the probability that stored UCB will be used is relatively high.

In general, we encourage mothers to donate UCB because it is a valuable resource for stem cell research and therapy. The government, clinicians and UCB banks should combine efforts to provide accurate and scientific counselling on this subject, increase the willingness of mothers to preserve UCB.

### Conclusions

The results showed that mothers with higher education or those having better occupations are more likely to preserve their UCB in China. These mothers have related knowledge and understand the importance of the preservation and they could more readily afford the relatively high cost. The government, clinicians and UCB banks should combine efforts to take measures such as increasing public knowledge of the importance of UCB preservation and decreasing the high cost for its storage will most likely increase the frequency of UCB preservation which will further benefit stem cell research and therapy.

## Supporting Information

S1 File The standardized interview procedure. (PDF)

S1 Table Statistical data. (XLSX)
