# Supplementary material for: Factors That Influence a Mother’s Willingness to Preserve Umbilical Cord Blood: A Survey of 5120 Chinese Mothers
Source: PLoS One. 2015 Dec 9;10(12):e0144001. doi: 10.1371/journal.pone.0144001 (PMC4674096; doi:10.1371/journal.pone.0144001)
Supplement: S1 File — (PDF) [file pone.0144001.s001.pdf]

# Interview procedure

(Oral questions and answers)

**Date:**

**The main topics discussed with the interviewee were as follows:**

**1) Personal information, including name, old, delivery time, occupation(s), and level of education.**

**2) Whether the patient had any knowledge of the preservation of UCB.**

**If not, an elaborate introduction of the principles, meaning and expenses of UCB preservation was provided, especially for use in stem cell therapy.**

**3) Whether the patient was willing to store her UCB.**

**4) If the patient wished to save her UCB, record it.**

If not, what were their greatest concerns?

① the safety of the preservation?

② consider it irrelevant?

③ high cost or other reasons?
